# Dynamic Expression of Follicle-Stimulating Hormone and Estrogen mRNA Receptors Associated with microRNAs *34a* and *-let-7c* in Canine Follicles during the Estrous Cycle

**DOI:** 10.3390/ani14020214

**Published:** 2024-01-09

**Authors:** Monica De los Reyes, Phillip Dettleff, Jaime Palomino, Oscar A. Peralta, Ana Vergara

**Affiliations:** 1 Laboratory of Animal Reproduction, Faculty of Veterinary Sciences, University of Chile, Santiago 8820000, Chile; ana.vergara@ug.uchile.cl; 2 School of Veterinary Medicine, Faculty of Agronomy and Natural Systems, Faculty of Biological Sciences and Faculty of Medicine, Pontifical Catholic University of Chile, Santiago 8320165, Chile; phillip.dettleff@uc.cl (P.D.); oscar.peralta@uc.cl (O.A.P.); 3 School of Veterinary Medicine, Faculty of Medical Sciences, Bernardo O’Higgins University, Santiago 8370993, Chile; jaime.palomino@ubo.cl

**Keywords:** dog, gene expression, ovarian cycle

## Abstract

**Simple Summary:**

The dynamic change in gene expression during follicular development is essential for oocyte maturation and ovulation. The genes encoding for estrogen receptor (*ESR2*) and follicle-stimulating hormone receptor (*FSHR*) play crucial roles in ovarian follicular development. Gene expression is subjected to different regulatory mechanisms, including microRNAs, which have been demonstrated to regulate specific target genes. No information is currently available on the expression of miRNAs in canine ovarian follicles; therefore, the objective of this study was to evaluate the expression of miRNAs *cfa-miR-34a* and *cfa-let-7c* predicted against *FSHR* and *ESR2*, respectively, related to their target gene expression in follicular cells throughout the estrous cycle in this species. The genes and miRNAs were evaluated using quantitative PCR analyses. Each miRNA and its target genes were expressed in all estrous phases. A tendency for an inverse relationship was observed between the expression of *miR-34a* and *FSHR* only in anestrus, while an inverse correlation was found between *miR-let-7c* and *ESR2* during the entire cycle. The differential expression profile of *miR-34a* and *miR-let-7c* and their predicted target genes in canine ovarian follicles obtained in the present study suggests a role of these miRNAs in the negative regulation of *FSHR* and *ESR2* genes throughout the ovarian cycle.

**Abstract:**

The genes encoding for estrogen receptor (*ESR2*) and follicle-stimulating hormone receptor (*FSHR*) play crucial roles in ovarian follicular development. This study aimed to determine the expression levels of miRNAs predicted against *FSHR* and *ESR2* mRNAs in follicular cells related to their target genes during the estrous cycle in canines. Antral follicles were dissected from 72 ovaries following ovariohysterectomies. MiRNAs regulating *FSHR* and *ESR2* genes were selected from miRNA databases, and mature miRNA and mRNA expression profiling was performed using real-time polymerase chain reaction (PCR). The best miRNA for each target gene was selected considering the quantitative PCR (qPCR) performance and target prediction probability, selecting only miRNAs with a binding *p*-value of 1.0, and choosing *cfa-miR-34a* and *cfa-let-7c* for *FSHR* and *ESR2*, respectively. The expression levels comparing the different phases of the estrous cycle were evaluated using ANOVA. Pearson correlations between the expression pattern of each miRNA and their target genes were performed. Each miRNA and its target genes were expressed in the granulosa cells in all estrous phases. *FSHR* remained low in anestrus and proestrus, increased (*p* < 0.05) to the highest level in estrus, and decreased (*p* < 0.05) in diestrus. *ESR2* showed the same trend as *FSHR*, with the highest (*p* < 0.05) expression in estrus and the lowest (*p* < 0.05) in anestrus and proestrus. A tendency for an inverse relationship was observed between the expression of *miR-34a* and *FSHR* only in the anestrus phase, while an inverse correlation (r = −0.8) was found between *miRNA-7c* and *ESR2* (*p* < 0.01). The expression profile of *miR-34a* and *miR-let-7c* and their predicted target genes of dog ovarian follicles throughout the estrous cycle observed in this study suggest a role in the transcriptional regulation of *FSHR* and *ESR2*, which is the first evidence of the involvement of these miRNAs in the canine follicular function.

## 1. Introduction

Ovarian follicular development is controlled by complex interactions between hormones produced in the hypothalamus, pituitary gland, and ovaries. Follicle-stimulating hormone (FSH) is secreted by gonadotrophs of the anterior pituitary gland in response to gonadotropin-releasing hormone (GnRH) [1] and acts upon its receptor follicle-stimulating hormone receptor (FSHR), which is a member of the rhodopsin receptor family of G protein-coupled receptors [2], stimulating follicular cells to produce steroids [3]. FSHR activation is necessary for the hormonal functioning of FSH and thus, it is crucial for follicular development. This receptor is encoded by the *FSHR* gene, and its expression has been reported in follicles of different species [4,5], including canines [6,7,8]. Although fluctuations in FSH circulating levels display temporal patterns, the changes in FSHR expression throughout the estrous cycle has not been previously described in canines.

On the other hand, estrogens (E2), which are produced by follicle cells, act directly by binding with estrogen receptors alpha (ERα) and beta (ERβ), which are ligand-activated transcription factors, and indirectly by activating plasma membrane-associated E R intracellular signaling [9,10]. Estrogen receptors alpha and beta subtypes are encoded by *ESR1* and *ESR2* genes, respectively. Predominantly expressed by granulosa cells [11], ERβ is associated with follicle growth and ovarian responsiveness in canines [12]. This receptor is essential for gonadotropin-induced steroidogenesis and gametogenesis [13,14]. In addition, ERβ regulates gonadotropin secretion acting in GnRH neurons [15].

Endocrine regulation of ovarian function involving FSH and E2 defines the follicular phase before ovulation [16]. The actions of both hormones depend on their receptor expression to perform their functions. At the same time, receptor mRNA expression strongly depends on the post-transcriptional modifications to which they are subjected [2]. Increasing evidence corroborates that miRNAs regulate ovarian function through their actions in ovarian cells, such as granulosa and cumulus cells [17,18], and oocytes [19]. MicroRNAs are the most abundant class of small RNAs in the ovary [20]. They are short, non-coding RNA molecules of approximately 17–22 nucleotides that have emerged as critical post-transcriptional regulatory RNA molecules [21]. MicroRNAs regulate the gene expression of target genes by binding to the 3′-untranslated regions (3′-UTR) of mRNAs, inhibiting their translation and/or causing their degradation [22,23], or, in minor proportion, by enhancing translation at the post-translation stage [24]. The effects of FSH on ovarian cell proliferation and estrogen levels could be mediated by several ovarian miRNAs [25,26]. Some studies have reported differences in miRNA expression during different ovarian stages; thus, dynamic changes in the profiles of different miRNAs have been described during follicle development in mice [27] as in other species [28,29]. Therefore, several follicular functions could be developmentally regulated by miRNAs throughout the estrous cycle. The understanding of the mRNA–miRNA relationship will provide insights into the mechanisms of normal follicle development, further providing the possibility of new reproductive techniques and treatments in canids. However, no information is currently available on the expression of miRNAs in canine ovarian follicles. Studies on other species are only sometimes homologous to dogs because the canine estrous cycle is not comparable to the cycles of other mammals due to its length and many peculiarities. Therefore, this preliminary study aimed to evaluate the expression of miRNAs predicted against *FSHR* and *ESR2*, related to their target genes in follicular cells, comparing different phases of the estrous cycle in canines.

## 2. Materials and Methods

### 2.1. Animals

Ovaries were collected from 36 non-pregnant mixed-breed canine females (aged 1–4 years) after routine ovariohysterectomies at veterinary centers. The phases of the estrous cycle were confirmed based on the progesterone measurement of blood samples obtained during neutering.

Animal procedures were performed following the guidelines established by the Animal Care Committee at the University of Chile and the Chilean National Agency for Research and Development (ANID), Ministry of Sciences and Technology (Number 21485—VET—UCH). Informed consent was obtained from the dog tutors.

### 2.2. Ovaries Processing and Follicles Isolation

The ovaries were kept in saline solution (NaCl 0.9%) at 4 °C and transported to the laboratory within 20–30 min. Each ovary was rinsed in saline solution thrice, and the adipose and connective tissues were removed and placed in Petri dishes (Falcon; Becton Drive, Biosciences. Franklin Lakes, NJ, USA), in PBS, pH 7.4, supplemented with 5% (*v*/*v*) heat-inactivated fetal calf serum (FCS) [30].

Ovaries without any visual abnormalities or cystic formation were selected for the experiments and classified in each estrous phase based on ovarian structures on the surface (follicles and corpus luteum) [31] and serum progesterone concentrations using the Minireader for Canine Progesterone (Minitube, Tiefenbach, Germany), and the specific test kit for progesterone (#219001110; Minitube). The values of progesterone for each stage of the cycle were based on previous studies. Proestrus, 0.2–2 ng/mL; estrus 2–18 ng/mL; diestrus > 20 ng/mL and anestrus < 0.19 ng/mL [31,32].

Individual antral follicles free of ovarian tissue were dissected using stereomicroscopes. Follicular cells from each antral follicle were retrieved manually using a 1 mL-gauge needle and a fine-tipped glass pipette after a puncture to release the intra-follicular contents. The method used to isolate granulosa cells was described in our previous studies [32,33]. In brief, the cumulus–oocyte complexes were discarded, and the aspirated granulosa cells and the follicular fluid were transferred to a conical tube and washed three times in PBS by centrifugation at 300× *g* for 10 min using an Eppendorf Centrifuge 5415 D (Eppendorf, Hamburg, Germany) at 22 °C.

The follicular cell pellets were transferred to RNAlater (Invitrogen, Carlsbad, CA, USA) in tubes and kept in pools. The classification of the estrous phase (anestrus, proestrus, estrus, and diestrus) of each sample was recorded prior to pooling and subsequently stored at −20 °C until total RNA extraction. Three different cell pools were used for each estrous phase, each containing granulosa cells from around 40–60 antral follicles.

### 2.3. microRNA Selection

As one miRNA could regulate more than one mRNA through miRNA–mRNA interactions, and one mRNA could be regulated by several miRNAs [34], we selected and tested specific miRNAs for the target genes. Thus, miRNAs regulating *FSHR* mRNA (*cfa-miR-8900*; *cfa-miR-34c*; *cfa-miR-34a*) and *ESR2* (*cfa-miR-8881*; *cfa-miR-8837*; *cfa-let-7c*) were selected from the *Canis familiaris* miRNAs databases available in the public microRNA databases miRbase [35] and miRWalk [36] (Table 1). The selection was based on the binding site-predicted targets available in the databases, based on the TarPmiR algorithm. From the predicted miRNAs targeting *FSHR* and *ESR2*, we considered the predicted probability of miRNAs and target mRNA considering the binding *p*-value (higher values represent a higher probability of being the target site) by evaluating three candidate miRNAs per target gene.

### 2.4. RNA Isolation, Primers, and qPCR Analysis

For miRNA and mRNA expression assessment, total RNA was extracted using the Gene JET RNA Purification Kit (#K0731, Thermo Scientific, Eugene, OR, USA), according to the manufacturer’s instructions. The concentration and purity of the total RNA were determined using an Epoch spectrophotometer (Epoch, Biotek Industries, Highland Park, IL, USA). The total RNA was stored at −80 °C.

The synthesis of cDNA from miRNA was performed using the Affinity Script qPCR cDNA synthesis kit (Agilent Technologies, Santa Clara, CA, USA) following manufacturer’s instructions. The cDNA concentration was assessed using the Epoch spectrophotometer (Epoch, Biotek Industries, Biotek Instrument, Winooski, VT, USA). We selected reference miRNAs previously validated in *Canis familiaris* to perform the miRNA expression analysis. Six miRNA-specific forward and universal reverse primers were used to amplify the mature miRNAs. Two reference miRNAs *cfa-miR-16* [37] and *cfa-miR-26a* [38] (Table 1) were the most stable reference miRNAs and were selected as normalizers for relative quantification of miRNA expression levels. Primers for amplifying canine miRNAs were designed using available sequences from previously described databases, or from publications regarding reference genes.

For *FSHR* mRNA, canine-specific primers were designed under standard procedures using information from GenBank sequences to *Canis familiaris*. For *ESR2*, canine-specific primers were used according to Goncalves et al. [39]. β-actin (*ACTB*) and *H2A* histone (*H2A*) were used as normalized reference genes, consistent with previous studies [31,40] (Table 2).

Quantitative expressions of miRNAs and mRNA were assessed with an Eco Real-Time PCR System Model EC-100-1001 (Illumina^®^, San Diego, CA, USA) using qPCR miRNA Master Mix (Agilent Technologies, Santa Clara, CA, USA) following the manufacturer’s protocols. For mRNA assessment, cDNA was obtained using the enzyme conjugate Affinity Script cDNA Synthesis Kit (Agilent Technologies, Santa Clara, CA, USA) and the cDNA concentration was determined using an Epoch spectrophotometer (Epoch, Biotek Industries). For mRNA and miRNA analysis, control samples, without reverse transcriptase, 10 ng of cDNA template, or primers, were included in duplicate in each plate. Data were analyzed using the ΔΔCT method of relative quantification.

### 2.5. Statistical Analysis

Multiple comparisons of the relative expression levels of miRNA and mRNAs in follicular cells and reproductive stage were analyzed by ANOVA using the InfoStat Professional Program, Version 2018, Cordoba, Argentina. The data were transformed into a normal distribution before applying the analysis. Significant differences among means were evaluated using the Duncan’s test.

The possible association between the expression pattern of each miRNAs and its target genes throughout the follicular development was evaluated using Pearson’s correlation coefficient.

All values were considered significantly different for *p* ≤ 0.05.

## 3. Results

The best miRNA for each target gene (*ESR2* and *FSHR*) was selected considering qPCR performance (low ct value and specific melt curve), and target prediction probability, selecting only miRNAs with a binding *p*-value of 1.0, with *cfa-miR-34a*, targeting the coding sequence in the position 1390 to 1438 of the *FSHR* gene, and *cfa-let-7c* (lethal-7), targeting the 3′ UTR sequence in the position 1915 to 1933 of the *ESR2* gene.

The expression of each miRNA and its target genes was observed in the granulosa cells at all reproductive cycle stages, confirming their presence in canine follicles. The relative abundance of *FSHR* and *ESR2* transcripts and the miRNAs *cfa-miR-34a* and *cfa-let-7c* exhibited specific-stage variations in follicular cells.

**Figure 1:**
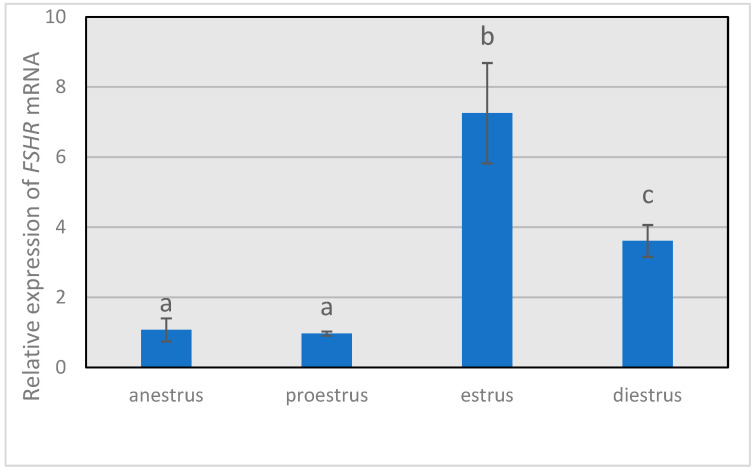
Gene expression levels of *FSHR* throughout the different phases of the estrous cycle. *FSHR* mRNA expression levels relative to the housekeeping genes β-actin RNA (*ACTB*) and histone H2A (*H2A*). Different letters above the bars indicate significant differences (*p* < 0.05).

**Figure 2:**
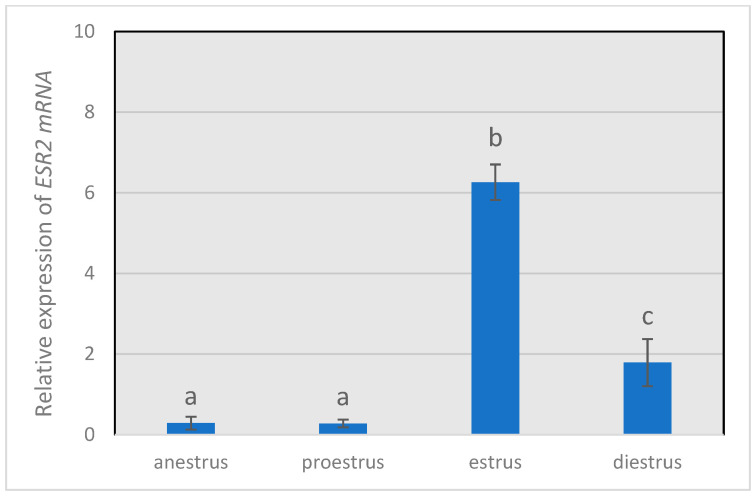
Gene expression levels of *ESR2* throughout the different phases of the estrous cycle. Gene expression levels of *ESR2* relative to those of the housekeeping genes β-actin RNA (*ACTB*) and histone H2A (*H2A*). Different letters above the bars indicate significant differences (*p* < 0.05).

The relative expression of *FSHR* (Figure 1) remained low during anestrus and proestrus, increasing (*p* < 0.05) to the highest value in the estrus phase and decreasing (*p* < 0.05) during diestrus, but with a higher (*p* < 0.05) expression level than that observed in anestrus or proestrus. The same trend was shown by *ESR2* transcript (Figure 2), with the highest (*p* < 0.05) relative abundance in estrus and the lowest values (*p* < 0.05) in granulosa cells from anestrus and proestrus.

**Figure 3:**
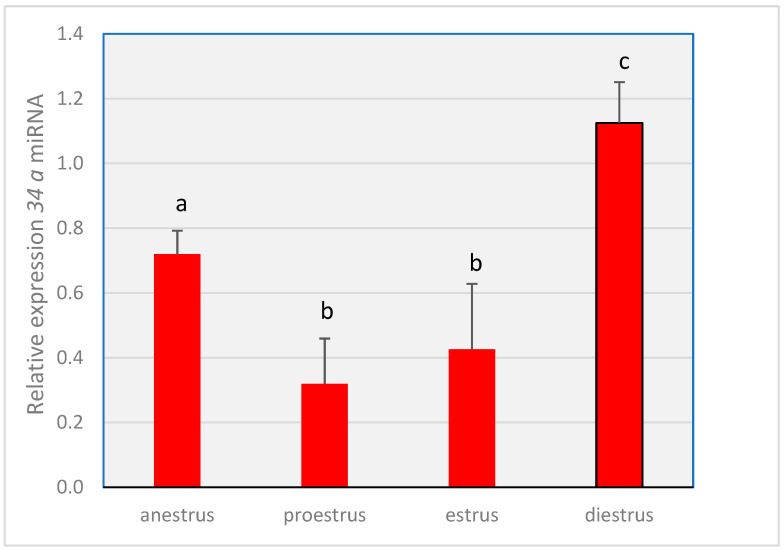
Gene expression levels of *34a miRNA* throughout the different phases of the estrous cycle. The miRNA expression of *miR-34a* relative to those of the reference miRNAs *cfa-miR-16* and *cfa-miR-26a.* Different letters above the bars indicate significant differences (*p* < 0.05).

**Figure 4:**
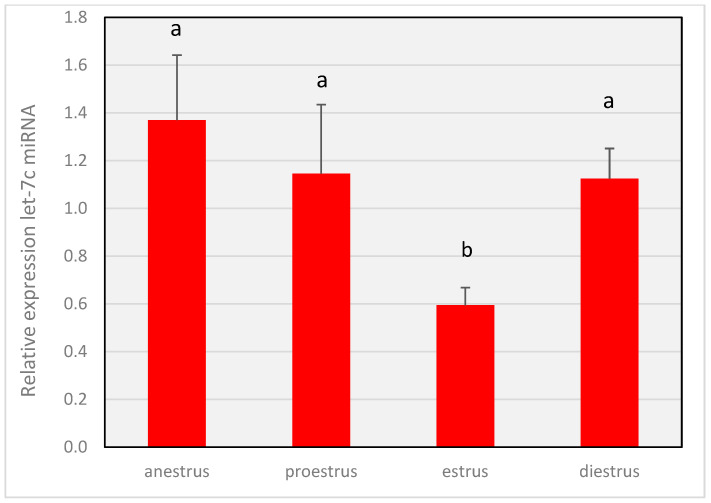
Gene expression levels of *miR-let-7c* throughout the different phases of the estrous cycle. The miRNA expression of *miR-let-7c* relative to those of the reference miRNAs *cfa-miR-16* and *cfa-miR-26a.* Different letters above the bars indicate significant differences (*p* < 0.05).

Differences (*p* < 0.05) were observed in the expression of both *miRNA-34a* (Figure 3) and *MiR-let-7c* (Figure 4) throughout the cycle. The expression level of *miR-34a* in proestrus and estrus was lower (*p* < 0.05) compared to that in anestrus and diestrus, with the highest values (*p* < 0.05) observed in the diestrus phase. *MiR-let-7c* showed a decrease (*p* < 0.05) in relative expression during the estrus phase compared to the other three stages, where the relative abundance was similar (*p* > 0.05).

**Figure 5:**
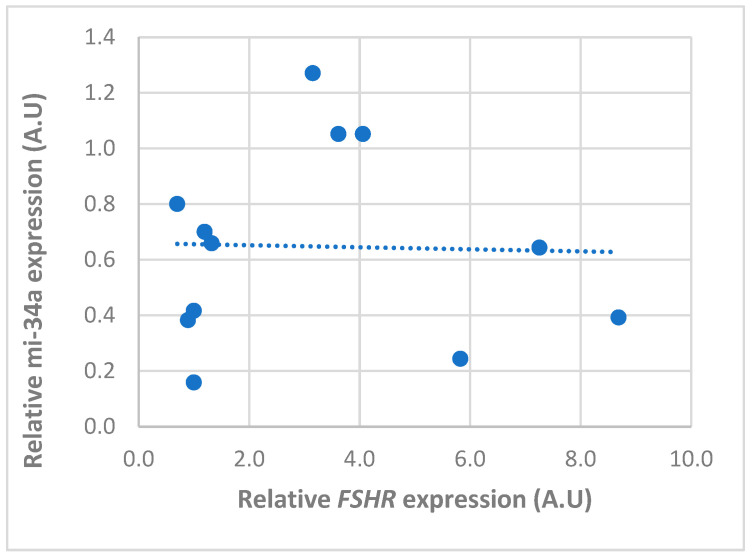
Pearson correlation between the relative expression of *miR-34a* and *FSHR* mRNA in follicular cells obtained from follicles in different phases of the canine estrous cycle. Dots represents the normalized expression level of each gene in A.U (arbitrary unit) and dotted line represent the trend line. No significant correlation was observed throughout the whole cycle (r = −0.06).

**Figure 6:**
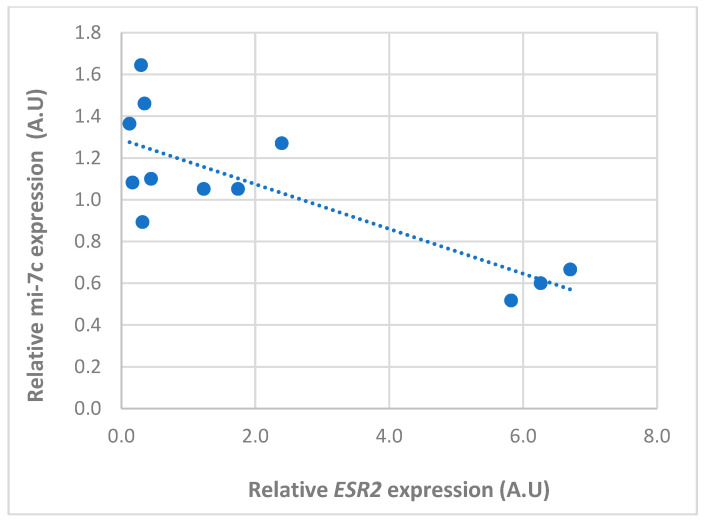
Pearson correlation between the relative expression of *miR-let-7c* and *ESR2* mRNA in follicular cells obtained from follicles in different phases of the canine estrous cycle. Dots represents the normalized expression level of each gene in A.U (arbitrary unit) and dotted line represent the trend line for correlation. An inverse and significative correlation (r = −0.8) was found between *miRNA-7c* and *ESR2* throughout the estrus cycle.

There was a tendency for an inverse relationship between the expression of *miR-34a* and its target gene (*FSHR*) only in the anestrus phase (Figure 5). However, no significant correlation was observed throughout the whole cycle (r = −0.06), whereas an inverse and significative correlation (r = −0.8) was found between *miRNA-7c* and *ESR2* (Figure 6; *p* < 0.01).

## 4. Discussion

This study demonstrated for the first time the expression of two miRNAs predicted against two essential genes, *FSHR* and *ESR2*, involved in follicular and oocyte development and their temporal abundance concerning their target genes in ovarian follicular cells in canines.

Although *FSHR* and its encoded receptors have been reported in canine granulosa cells [6,41], its variation throughout the estrous cycle has not been previously described in this species. The different *FSHR* expression patterns found during the estrous phases in the present study showed cyclic changes in growing follicles, implying that *FSHR* expression could be developmentally and hormonally regulated in dogs, like in other species [2,42]. Notably, the lowest *FSHR* mRNA expression levels were observed during the anestrus and proestrus phases. During the anestrus, the concentration of circulating FSH is the highest in the dog estrous cycle [43], although, without changes in the expression of its receptor (FSHR) [7]. Coincidentally, it has been demonstrated that FSH stimulation can inhibit *FSHR* transcription in Sertoli cells [44], involving self-regulation between hormone levels and the gene expression of its receptor. However, *FSHR* expression involves various other regulatory mechanisms that affect its stability and translation efficiency [42]. The *FSHR* is a target gene of different miRNAs, as demonstrated for *miRNA*-125 in porcine granulosa cells [29], and *miRNA-3* and *miRNA-143* in bovine granulosa cells [26]. We analyzed the expression pattern of *cfa-miRNA-34a* (*miR-34a*) in canine granulosa cells, which was predicted to regulate canine *FSHR*. The expression of *miR-34a* was higher in the anestrus and diestrus phases than in the other phases, which was consistent with the low level of *FSHR* relative expression in anestrus, when FSH concentration is high, but not in the diestrus stage, when the circulating FSH is low. MicroRNA *34a*, as a member of the miR-34 family (*miR-34a/b/c*, *miR-34s*) [45], has been proposed to control essential ovarian functions and negatively regulate the local translation of *FSHR*. In porcine ovarian cells, FSH promotes the expression of *miR-34a* [46], indicating that *miR-34a* may be involved in controlling ovarian functions; as both, *miR-34a* and FSH are synergists in their actions on follicular cell activity. Therefore, the influence of *miR-34a* on *FSHR* may depend on FSH levels which are high during canine anestrus. This could explain the trend towards an inverse relationship between *cfa-miRNA-34a* and its target gene only in anestrus, and the lack of significance in the correlation between *cfa-miRNA-34a* and *FSHR* abundance throughout the other phases of the estrous cycle.

Granulosa cells express mainly ERβ [47], and the ovary is the primary site associated with the highest level of ERβ [48]. Therefore, ERβ seems to be a critical mediator of estradiol signaling in granulosa cells orchestrating folliculogenesis. In a mouse model, *ESR2* mRNA expression has been associated with ovarian responsiveness to gonadotropins [49]. The present findings showed that *ESR2* mRNA was highly expressed in follicular cells during the estrus period, with very low transcript abundance prior to this stage. In other species, *ESR2* in follicles is mainly expressed during the follicular phase [50] and is possibly involved in regulating the ovulation process during the estrus stage. In *ESR2*-knockout mice, luteinizing hormone receptors become insufficient for ovulation [45]. Furthermore, it has been reported that the pre-ovulatory estradiol surge in dogs probably triggers the pre-ovulatory peak in LH [33,51]. Therefore, estradiol could influence the ovulation process via *ER*β-*ESR2* mediation [12]. However, the precise mechanisms by which *ESR2* regulates the estrous cycle and ovulation mechanisms are not yet fully understood.

MicroRNAs have been involved in the regulation of estrogen production in granulosa cells [52]. *MiR-let-7c* is one of the earliest identified miRNAs [53], and its family members are highly conserved across species and the most abundant miRNAs in the ovary [54,55], suggesting that they have fundamental roles in reproductive physiology. Here, *miR-let-7c* was observed during all phases of the canine estrous cycle, which agrees with the presence of *miR-let-7c* in the ovine [56] and caprine [57] granulosa cells. The lowest *miR-let-7c* expression level was observed in the estrus phase, while the relative gene expression of *ESR2*, the predicted target of *miR-let-7c*, was the highest. In contrast, in other phases of the cycle, the expression of *miR-let-7c* was higher, whereas that of the *ESR2* gene was lower. Therefore, *miR-let-7c* was inversely related to *ESR2* expression, consistent with previous studies on breast cancer stem cells [58]. *MiR-let-7c* can regulate *ER*β presence by directly targeting its mRNA *ESR2*, decreasing its expression [58], or affecting signaling pathways involved in *ESR2* expression [58]. Further research is necessary to decipher the complex regulatory mechanisms underlying *ESR2* and *miR-let-7c* expression and their roles in reproductive physiological processes in canines.

## 5. Conclusions

The differential expression profile of *miR-34a* and *miR-let-7c* and their predicted target genes in canine ovarian follicles throughout the estrous cycle obtained in the present study suggest a role in the transcriptional regulation of *FSHR* and *ESR2* genes, which represents the first evidence of the involvement of these miRNAs in the canine follicular function, opening the possibility of investigating their future use in different reproductive treatments in this species. Advanced research on miRNAs in the canine ovary will help to understand the mechanisms and regulation involved.

## Figures and Tables

**Table 1:** List of miRNA Primers sequences for RT-qPCR.

Target Gene	miRNA	Primer
*ESR2*	*cfa-miR-8881*	UUUGUUUUCUCUGGUUCUGUACC
	*cfa-miR-8837*	UUCUUGCUGGAGUCCGGUUGUCU
	*cfa-let-7c*	UGAGGUAGUAGGUUGUAUGGUU ***
*FSH-R*	*cfa-miR-8900*	UAGGACUUUAAUGGCUGGAGAGA
	*cfa-miR-34c*	AGGCAGUGUAGUUAGCUGAUUGC
	*cfa-miR-34a*	UGGCAGUGUCUUAGCUGGUUGU. ***
ref miRNAs	*cfa-miR-16*	5′-UAGCAGCACGUAAAUAUUGGCG-3′
ref miRNAs	*cfa-miR-26*	5′-UUCAAGUAAUCCAGGAUAGGCU-3′

*** Selected miRNA considering qPCR performance and target predicted probability with binding *p*-value of 1.0.

**Table 2:** Sequences of primers for references genes used in this study and *FSH-R*, *ESR2* genes evaluated for qRT-PCR analysis.

Gene	Sequence 5′-3′	Accession Number	Amplicon	Tm °C	Efficence
*ACTB*	F:ATTGTCATGGACTCTGGGGATG	AF021873.2	191 bp	56.7	1.99
	R:TCCTTGATGTCACGCACGAT				
*H2A*	F:AGTACCTGACGGCCGAGAT	XM545419.4	245 bp	59.6	1.97
	R:AGGGCAAATCAATCCAGAGA				
*ESR2*	F: TTCTATAGCCCTGCTGTGATGAAT	AF389885.1	204 bp	60.0	2.01
	R: ATTATGTCCTTGAATGCTTCTTT				
*FSH-R*	F:AACTCATTTGGCCATCCTTG	NC051814.1	212 bp	60.0	1.98
	R:TGACTGCACCTTAGGCAGTG				

Abbreviation: *FSHR*, follicle-stimulating hormone gene receptor. *ESR2*, Estrogen gene receptor beta. *ACTB*, β-actin and *H2A*, histone genes.

## Data Availability

The data of this study are available on request from the corresponding author.
